# Traveller mothers: Obstetric and neonatal outcomes in an Irish maternity unit

**DOI:** 10.1002/ijgo.70663

**Published:** 2025-11-22

**Authors:** Nessa Hughes, Stephen W. Lindow, Lynsey Kavanagh, Barbara Burke, Lyndsay Creswell, Emma McNamee, Mark P. Hehir, Michael P. O'Connell

**Affiliations:** ^1^ Obstetrics and Gynaecology The Coombe Hospital Cork St Dublin Ireland; ^2^ Pavee Point Traveller and Roma Centre Dublin Ireland

**Keywords:** consanguinity, low birthweight, pregnancy, prematurity, smoking, Traveller

## Abstract

**Objective:**

Travellers' ethnicity was only formally acknowledged by the Irish State in 2017. They experience persistent racism and discrimination, resulting in poorer outcomes in terms of health, education, employment, and accommodation. Previous studies have reported higher rates of infant mortality and stillbirths among Traveller mothers. This study is a retrospective review examining pregnancy and neonatal outcomes for Traveller mothers compared to the general Irish population.

**Methods:**

The hospital database was interrogated for results of singleton pregnancies between January 1 2013 and December 31 2023 inclusively where mothers self‐identified as Travellers and variables analyzed.

**Results:**

Traveller women become mothers at a younger age (25 vs. 33 years, *P* < 0.001) and are more likely to be married (72% vs. 58.5%, *P <* 0.001) with higher median body mass index measurements (26 vs. 24.8, *P <* 0.001). There are increased rates of prematurity (18.6% vs. 13.3%, *P <* 0.001) and delivery of a low weight baby (8.4% vs. 5.1%). They are more likely to smoke in pregnancy (45.6% vs. 8.9%, *P <* 0.001) and be related to the father of the baby (24% vs. 0.88%, *P <* 0.001). Smoking and drug use were found to be independent risk factors for prematurity (odds ratio [OR] 1.734, 95% confidence interval [CI] 1.606–1.872, *P <* 0.001 and OR 1.710, 95% CI 1.316–2.223, *P <* 0.001, respectively) but ethnicity was not (OR 1.198, 95% CI 0.989–1.451, *P* = 0.065).

**Conclusion:**

Being a Traveller itself or consanguinity was not a factor in premature delivery but smoking and drug use in pregnancy was associated with premature delivery. This is an opportunity to offer support to Traveller women and potentially to improve neonatal outcomes.

## 1 INTRODUCTION

Travellers are an Irish minority group whose ethnicity was only formally acknowledged by the Irish state in 2017.^1^ The All‐Ireland Traveller Health Study, 2010 (AITHS), a large national Irish study, established the Traveller population at 36 224 in the Republic of Ireland.^2^ According to Census 2022, 32 949 Travellers self‐identified their ethnicity, equating to approximately 0.64% of the general population.^3^ However, the most recent annual Traveller family count in 2021 undertaken by another Irish government department reports 12 367 Traveller families in Ireland or 49 468 Travellers (based on Census 2022 average family size of four).^4^

Although Ireland is considered one of the wealthiest European countries, Traveller health outcomes are exceptionally poor, with the Traveller population pyramid similar to that of a developing country, reflecting higher fertility and higher mortality rates across all ages and genders with shorter life expectancy.^5^ This is echoed in AITHS, which observed significant health inequalities when compared to the majority population in Ireland. It found that Traveller women have three times the mortality rate, live on average 11.5 years less and have a suicide rate that is five times higher than women in the general population. It also found that Traveller infant mortality was estimated at 14.1 for every 1000 live births, almost four times the rate found in the general population.^2^

The same study showed that almost 98% of Traveller women accessed antenatal care, but 6.1% of Irish Travellers delivered a baby <2.5 kg versus 3.7% in the general population.^2^ Rates of early pregnancy loss, low birth weight, and preterm delivery are all quoted to be higher among Traveller women. The findings also show that Traveller mothers have a shorter birth gap between pregnancies and higher parity and stillbirth rates compared to the general population. As seen with other indigenous, marginalized, and minority ethnic groups of women internationally, as in MBRRACE in the UK and nationally in Ireland, maternal mortality and morbidity is disproportionate.^6^, ^7^

In 2021, the European Union Agency for Fundamental Rights found that 65% of Travellers reported experiencing discrimination, one of the highest reported rates within the six European countries it surveyed.^8^ The AITHS study confirmed this, with only 41% of Travellers having complete trust in healthcare professionals compared to a trust level of 82% in the general population.^2^ As a result, Travellers experience poorer access, participation, and outcomes across all key social determinants of health. This means that as well as healthcare services, wider issues can affect health status, for example poor accommodation, homelessness, unemployment, poor educational attainment, discrimination, racism, and inequality in services.

Despite having formal access to the same health services, Travellers are not benefiting from advances in mainstream health compared to those in the majority population. Local action plans with community health workers and ongoing research aim to provide wider access and benefits to Traveller mothers through accessible and educational resources that can help bridge this gap.

## 2 OBJECTIVE

We aimed to conduct a retrospective review of singleton pregnancies at The Coombe Hospital for a 10‐year period between 2013 and 2023, examining pregnancy and neonatal outcomes for Travellers compared to the general Irish population.

## 3 PATIENTS AND METHODS

The Coombe Hospital is a tertiary, university teaching maternity hospital in Dublin, which anticipates approximately 7500 births per year. The unit maintains a database of all new patients' histories taken at the time of the first antenatal visit. This dataset is updated at the time of delivery with pregnancy, neonatal, and delivery details.

The data collected includes the patient's self‐identified ethnicity. This includes a general ethnicity question for both the woman and her partner and a specific separate question that asks if they self‐identify as a Traveller. Patients who answered yes were included in the Traveller cohort, in addition to those whose partner was a Traveller. Those who did not meet the above criteria were included the “all other” ethnicity cohort; this included women who identified as members of the Roma community who are sometimes considered synonymous with the Traveller population, as they are another traditionally nomadic group. Roma women are culturally and ethnically different, however, and were therefore not included in the Traveller group.

This hospital database was interrogated for results of singleton pregnancies between January 1 2013 and December 31 2023 inclusively, and all data was anonymized. Patients were coded as being of “Traveller” or “all other” ethnicities. Other variables were examined, including maternal age, body mass index (BMI), marital status, gravidity, parity, smoking status, drug and alcohol use in pregnancy, consanguinity, and preconceptual folic acid use. The study dataset was maintained in Microsoft Excel (Microsoft Corporation, Richmond, WA, USA), and statistical analysis was performed using IBM's SPSS Statistics for Windows 29.0 (IBM, Armonk, NY, USA). A representative from Pavee Point (LK), a national non‐governmental organization working with Travellers, was part of the study team to ensure there was input and representation from the Traveller community.

The study was approved by the Audit and Quality Advisory (AQuA) group at the Coombe Hospital, who give approval to audit hospital data.

## 4 RESULTS

During the study period, 1723 (2.1% of total) women self‐identified as a Traveller and 81 850 did not. Normality testing on maternal age at delivery, BMI, gestation at delivery, birthweight and last recorded hamoglobin revealed that all parameters were not normally distributed (Kolmogorov–Smirnov test statistic 0.077, 0.099, 0.201,0.057 and 0.034, respectively, *P <* 0.001 in all analyses). Non‐parametric analysis was therefore undertaken. The Beutler test was performed in 1459/1579 (92.4%) pregnant Traveller women.

### 4.1 Maternal demographics and characteristics

The characteristics of Traveller and other ethnicity women are described in Table 1. Traveller women were more likely to be of Irish nationality, white and married. They became mothers at a younger age and had higher median BMI measurements. Traveller women were significantly more likely to be smokers in pregnancy (45.6% vs. 8.9%, *P <* 0.001) and have ever smoked (66% vs. 42.3%, *P <* 0.001). Traveller women were more likely to have a baby with another Traveller (87.1% vs. 0.3%, *P <* 0.001) and more likely to be related to the father of the baby than non‐Traveller women (24% vs. 0.88%, *P <* 0.001).

**TABLE 1:** Demographic information for participants.

	Traveller *n* = 1723	All others *n* = 81 850	Odds ratio (95% confidence interval)	Significance (*χ* ^2^ analysis unless stated)
Median (range) age (years)	25 (16–44)	33 (14–54)		Mann–Whitney *U* −38.8 *P <* 0.001
Median (range) BMI	26 (15.5–59.4)	24.8 (10.4–82.6)		Mann–Whitney *U* 7.9 *P <* 0.001
Irish nationality *n* (%)	1410 (81.9%)	57 182 (68.9%)	1.9 (1.7–2.2)	115.4 *P <* 0.001
White (%)	1717 (99.7%)	69 669 (85.1%)	50 (22.4–111.5)	286.2 *P <* 0.001
Married *n* (%)	1241 (72%)	47 854 (58.5%)	1.8 (1.7–2.0)	128 *P <* 0.001
Primigravida^a^ *n* (%)	358 (20.8%)	25 494 (31.1%)	0.63 (0.56–0.70)	84.9 *P <* 0.001
Primipara^b^ *n* (%)	457 (26.5%)	33 312 (40.7%)	0.53 (0.47–0.59)	140.9 *P <* 0.001
Smoking ever *n* (%)	1137 (66.0%)	34 657 (42.3%)	2.6 (2.4–2.9)	385.4 *P <* 0.001
Current smoker in pregnancy *n* (%)	785 (45.6%)	7214 (8.9%)	8.7 (7.9–9.6)	2632 *P <* 0.001
Alcohol use in pregnancy *n* (%)	26/1028 (2.5%)	645/60 530 (1.1%)	2.4 (1.6–3.6)	20.1 *P <* 0.001
Drug use *n* (%)	26 (1.5%)	453 (0.6%)	2.8 (1.9–4.1)	27.0 *P <* 0.001
Partner has a history of drug use *n* (%)	53 (3.1%)	3261 (4.0%)	0.77 (0.59–1.0)	3.7 *P* = 0.56 (NS)^c^
Electronic cigarettes/vaping use *n* (%)	51/916 (5,6%)	1496/42 372 (3.5%)	1.7 (1.3–2.2)	12.5 *P <* 0.001
Preconception folic acid *n* (%)	317 (18.4%)	29 384 (35.9%)	0.40 (0.36–0.46)	225.6 *P <* 0.001
Unplanned pregnancy *n* (%)	564 (32.7%)	22 203 (27.1%)	1.3 (1.2–1.5)	26.8 *P <* 0.001
History of mental health problems *n* (%)	415 (24.1%)	18 579 (22.7%)	1.1 (0.97–1.2)	1.9 *P* = 0.17 (NS)
Homeless/refuge *n* (%)	9 (0.33%)	136 (0.17%)	3.2 (1.6–6.2)	12.4 *P <* 0.001
Domestic violence *n* (%)	37 (2.1%)	747 (0.9%)	2.4 (1.7–3.3)	27.6 *P<* 0.001
Father a blood relative *n* (%)	385/1603 (24%)	667/76 032 (0.88%)	35.7 (31.1–41.0)	6289 *P <* 0.001
Father a Traveller *n* (%)	1391/1597 (87.1%)	198/75 760 (0.3%)	2583 (2110–3162)	58 744 *P <* 0.001

^a^ Primigravida: pregnant for the first time.

^b^ Primipara: giving birth for the first time.

^c^ No significance [NS].

While variables such as alcohol use in pregnancy, vaping, homelessness and domestic violence were more common among Traveller women, and reached significance (*P <* 0.001), absolute numbers in each group were small. Traveller women were more likely to consider their pregnancy unplanned (32.7% vs. 27.1%, *P <* 0.001) and less likely to take periconceptual folic acid (18.4% vs. 35.9%, *P <* 0.001).

### 4.2 Pregnancy and neonatal outcomes

The pregnancy outcomes for both groups are described in Tables 2 and 3. There was a significantly higher premature birth rate (18.6% vs. 13.3%, *P <* 0.001) and a significantly lower median birthweight (3400 g vs. 3460 g, *P <* 0,001) in the Traveller group. Low birthweight was more common in the Traveller group, although the rate of large babies >4500 g was similar. The stillbirth rate was the same in both groups.

**TABLE 2:** Pregnancy and neonatal outcomes of participants.

	Traveller *n* = 1723	All others *n* = 81 850	Odds ratio (95% confidence interval)	Significance (*χ* ^2^ analysis unless stated)
Median (range) gestational age (w)	39 (22–44)	39 (22–44)		Mann–Whitney *U* −4.8 *P <* 0.001
Median (range) birthweight (g)	3400 (242–5350)	3460 (155–5840)		Mann–Whitney *U* −5.6 *P <* 0.001
Median (range) last recorded hemoglobin (g/dL)	11.5 (6.2–15.7)	12.1 (4.3–21.1)		Mann–Whitney *U* 19.1 *P <* 0.001
Premature births 22–37‐week gestation *n* (%)	321 (18.6%)	10 898 (13.3%)	1.5 (1.3–1.7)	41.0 *P <* 0.001
Gestational age breakdown of deliveries 22–27 weeks *n* (%)	16 (0.9%)	424 (0.5%)		42.4 *P <* 0.001
28–31 weeks *n* (%)	16 (0.9%)	531 (0.6%)		
32–37 weeks *n* (%)	289 (16.8%)	9943 (12.1%)		
38–45 weeks *n* (%)	1402 (81.4%)	70 952 (86.7%)		
Stillbirths *n* (%)	7 (0.4%)	290 (0.4%)	1.2 (0.54–2.43)	0.13 *P* = 0.72 (NS)^a^
Caesarean section *n* (%)	483 (25.1%)	26 001 (31.8%)	0.84 (0.75–0.93)	13.4 *P* < 0.001
Apgar <7 at 1 min *n* (%)	101 (5.9%)	3976 (4.9%)	1.2 (0.99–1.5)	3.6 *P* = 0.06 (NS)
Apgar <7 at 5 min *n* (%)	26 (1.5%)	601 (0.7%)	2.1 (1.4–3.1)	13.5 *P <* 0.001
PPH >500 mL *n* (%)	216/1217 (17.7%)	12 422/57 904 (21.5%)	0.79 (0.68–0.92)	9.7 *P <* 0.01
Normal neonatal examination *n* (%)	1640 (95.7%)	78 740 (96.7%)	0.76 (0.60–0.97)	4.9 *P <* 0.05

^a^ No significance [NS].

**TABLE 3:** Birthweight distributions, by ethnicity groups.

Birthweight (g)	Traveller *N* = 1723	All others *N* = 81 850	*χ* ^2^ analysis
<2500	144 (8.4%)	4183 (5.1%)	45.4 *P <* 0.001
2500–3499	489 (49.3%)	38 890 (47.5%)	
3500–4499	701 (40.7%)	37 512 (45.8%)	
>4500	29 (1.7%)	1265 (1.5%)	

To further assess this increased rate of preterm birth, a logistic regression analysis was conducted (see Table 4). All pregnancies for which complete data was available were included (*n* = 57 438). Smoking and drug use were found to be independent risk factors in this dataset (odds ratio [OR] 1.734, 95% confidence interval [CI] 1.606–1.872, *P <* 0.001 and OR 1.710, 95% CI 1.316–2.223, *P <* 0.001, respectively), and as noted above are more common in the Traveller cohort. Ethnicity, however, was not found to be an independent risk factor for preterm birth (OR 1.198, 95% CI 0.989–1.451, *P* = 0.065). This approaches significance, so a larger dataset or the inclusion of other confounding variables might affect this result.

**TABLE 4:** Logistic regression for preterm birth risk.

Variable	OR	95% confidence interval		Significance
		Lower	Upper	
Smoker	1.734	1.606	1.872	*P <* 0.001
Consanguinity	0.990	0.703	1.395	*P* = 0.955
Preconceptual folic acid	1.016	0.964	1.070	*P* = 0.559
Drug use	1.710	1.316	2.223	*P <* 0.001
Alcohol use	1.099	0.878	1.376	*P* = 0.409
Ethnicity	1.198	0.989	1.451	*P* = 0.065

Traveller women were noted to have a lower median last recorded hemoglobin in pregnancy. They were, however, less likely to have a caesarean section or a postpartum hemorrhage.

There was no difference found in APGAR scores <7 between the two groups at 1 minute. However, Traveller neonates were more likely to have a score <7 at 5 min. Additionally, while most infants had a normal neonatal examination, Traveller neonates were less likely to have a normal examination.

## 5 DISCUSSION

Most published population data for Irish Travellers is outdated, and given the clear health disparities shown in previous studies, it is clear that the available data requires revision. Literature reviews looking at discrepancies in their care and pregnancy outcomes emphasize the need for tailored care for marginalized groups.^9^

The present study shows that Traveller women become mothers at significantly younger ages. The median age for a pregnant Traveller woman was 6.5 years less than the national average of first‐time mothers in Ireland in 2022 (25 vs. 31.5 years).^10^ Although younger, they are more likely to be overweight and smokers, both of which are known to have negative effects on pregnancy. Higher rates of pregnancies that were considered unplanned were also reported, although it is not clear how this question is asked at booking and might have different interpretations. In addition, lower rates of preconceptual folic acid were taken among Travellers.

During pregnancy, smoking cessation should be discussed regularly given the high rates of smoking in pregnancy among these women (45.6%). These rates for Traveller women are like those in AITHS (48%) and a more recent 8‐year review of smoking in the Irish obstetric population (40.3%).^2^, ^11^ Adherence to smoking education is a potential area to reduce pregnancy smoking rates to a similar rate seen in the general population. Although the numbers were small, Traveller mothers were at higher risk of harsher conditions, with domestic violence, homelessness, and maternal illicit drug and alcohol use demonstrating greater prevalence. Ensuring there is appropriate and accessible safety screening for these identified vulnerable women is essential at every antenatal visit.

Infants born to Traveller mothers are more likely to be premature or low birthweight. In AITHS, they found that even after adjusting for both smoking and alcohol use, Traveller mothers were still more likely to have a low birthweight infant.^2^ The present data revealed that the significant associations with premature delivery were smoking and drug use. Traveller ethnicity or consanguinity were not independent factors. There is a clear issue, and a possible educational target, with smoking in pregnancy among Traveller women that could potentially improve neonatal outcomes for the group if addressed.

Previous studies quote that stillbirth rates are higher among Traveller women: 5% compared to 1.6% in Irish women.^12^ One Irish study estimated that 12% of Traveller women suffered one or more stillbirths.^13^ The stillbirth rate was not increased in our study. With evolving health care, stillbirth rates have declined significantly over the past 30 years in Ireland, with a study from another Irish hospital attributing some of this to a decrease in deaths to placental abruption and likely improved overall antenatal care and public health.^14^ Our findings present this as a positive trend for Traveller women too.

Both groups had similarly high rates of normal initial neonatal examinations. However, with lower APGAR scores at 5 min, this might reflect the complications that Traveller babies face in infancy. With infant mortality rates almost four times that of the general population, higher rates of some genetic conditions evident among some Traveller families might contribute to this.^2^, ^15^ Many of these are rare autosomal recessive disorders that are almost unique to this population. A catalogue of 91 rare disorders that occur in Irish Travellers has been published to help identify and, if possible, plan for support.^16^ As an example, one in 450 Traveller births will be affected by classical galactosemia, a disorder of galactose metabolism, compared to 1 in 36 000 of the general population.^17^, ^18^ This rare condition can be life‐threatening, and Traveller mothers are advised not to breastfeed during the first days of life until a definitive test, the Beutler test, returns with a negative result.^19^ In this study, 92% of Traveller babies had this test. Babies are not routinely offered this test otherwise. Consanguinity is reportedly more common among Travellers, but rates are not well described in recent data.^20^ Access to preconception and antenatal screening is becoming more available, but its uptake or benefit in the Traveller population has not been well studied. Education for healthcare professionals is also important to be able to provide accurate information for reproductive decisions and be aware of actual genetic risk versus their impression of it. This needs to be undertaken in a respectful and culturally appropriate manner.

Limitations of the study include missing demographic variables, including socioeconomic status and education, that might influence outcomes. These are not routinely asked at booking as they might be perceived as discriminatory or induce bias in healthcare provision. In addition, there is risk of underreporting with smoking or drug use in pregnancy and consanguinity given the negative associations. Maternal health issues were not analyzed and might be more prevalent in certain groups or contribute to outcomes. There would be benefit in undertaking an up‐to‐date national study using existing maternity datasets looking at access and outcomes for Traveller women and neonates given that 69.4% of Traveller mothers had births outside Ireland's capital city Dublin in 2011, where our hospital is based.^2^ There might be changes in the distribution of young Travellers in the country from the country toward cities. However, as a group they are mobile and data from one hospital might reflect a wider geographic distribution of Traveller women.

There is a need for the data to be updated. The AITHS was published over 10 years ago. With advancements in maternity care, change is likely present. Through local and national initiatives and action plans since, some improvements have already become evident, including increased participation in antenatal booking and maternity services and increased Traveller infant vaccination uptake.^21^ This is largely due to the work of Primary Health Care for Travellers Projects, which are partnership projects between the Irish Health Service Executive (HSE) and Traveller organizations that provide ongoing support for Traveller families on the ground and act as an interface between mainstream health services and Travellers. These projects operate with a peer‐led model that trains Travellers to work as community health workers, and this allows primary healthcare to be developed based on the Traveller community's own values and perceptions.

## 6 CONCLUSION

The pregnancy outcomes in Traveller women show an increase in the rate of prematurity, a lower median birthweight, and an increase in the prevalence of low birthweight. Traveller women are more likely to smoke in pregnancy and be related to the father of the baby. Being a Traveller itself or consanguinity was not a factor in premature delivery but smoking and drug use in pregnancy was associated with premature delivery. This is an opportunity to offer education regarding the risks of smoking and drug use and support to Traveller women who wish to reduce their risks and potentially to improve neonatal outcomes.

## 7 GLOSSARY

The Government of Ireland (2002) defined the Traveller community as follows: “Traveller community means the community of people who are commonly called Travellers and who are identified (both by themselves and others) as people with a shared history, culture and traditions, including historically, a nomadic way of life on the island of Ireland.”^22^

## AUTHOR CONTRIBUTIONS

The conceptualization of the study was by N. Hughes and SW. Lindow. Methodology included data curation by E. McNamee and formal analysis by SW. Lindow, B. Burke and L. Creswell. Writing of original draft and research of references by N. Hughes. All authors have read, critically reviewed, edited and agreed to the published version of the manuscript. Supervision by SW. Lindow, MP. Hehir, MP O'Connell.

## FUNDING INFORMATION

None.

## CONFLICT OF INTEREST STATEMENT

The authors have no conflicts of interest to declare.

## Data Availability

Data sharing is not applicable to this article as no new data were created or analyzed in this study.
